# On the Relationship Between Authentic Leadership, Flourishing, and Performance in Healthcare Teams: A Job Demands-Resources Perspective

**DOI:** 10.3389/fpsyg.2021.692433

**Published:** 2021-07-29

**Authors:** Pedro Marques-Quinteiro, Ana Margarida Graça, Francisco Antonio Coelho, Daniela Martins

**Affiliations:** ^1^William James Center for Research, ISPA – Instituto Universitário, Lisbon, Portugal; ^2^Division of Leadership, Organisations and Behaviour, Henley Centre for Leadership, Henley Business School, University of Reading, Henley-on-Thames, United Kingdom; ^3^Department of Administration and Postgraduate Program in Administration, University of Brasília, Brasília, Brazil; ^4^Social and Organizational Psychology Department, ISPA – Instituto Universitário, Lisbon, Portugal

**Keywords:** authentic leadership, job demands-resources model, performance, nurses, flourishing at work

## Abstract

This study integrates the job demands-resources model and authentic leadership theory to test the general hypothesis that authentic leadership is a job resource that enables flourishing and performance in healthcare teams. Furthermore, this article tests the hypothesis that the daily bed occupancy is a job demand that weakens this relationship. Participants were 106 nurses that were distributed across 33 teams from two hospitals. The results suggest that the authentic leadership of team leaders is positively related with subjective and objective team performance, but only when daily bed occupancy is low. Authentic leadership had no relationship with team flourishing, regardless of the daily bed occupancy. Our findings suggest that the extent to which authentic leadership is adequate to promote the performance of teams working in a hospital setting is sensitive to contextual boundary conditions. Leading authentically might only be effective under specific circumstances.

## Introduction

Recently, the COVID-19 pandemic has exposed healthcare personnel to tremendous levels of job-related stress. The novelty of the virus, the uncertainty of the most adequate medical procedures, and the unprecedent daily bed occupancy were amongst the main contextual factors that challenged the performance and well-being of healthcare nurses (Zhang et al., 2020). To thrive in such adverse conditions, nurse teams were heavily dependent on the leadership capacity of those in charge (e.g., Hofmeyer et al., 2020). Leadership is at the heart of effective teamwork and the extent to which team leaders enable team effectiveness outcomes such as performance, viability and satisfaction has been extensively supported across meta-analyses and literature reviews (Avolio et al., 2009; Kozlowski et al., 2009). Team leadership has been found to be particularly important in complex work environments such as healthcare, where the performance and the well-being of health professionals such as nurses are often pushed to the limit (e.g., Landeweerd and Boumans, 1994; Wong et al., 2013).

One promising leadership construct that has the potential to enable a high-quality work environment in healthcare is authentic leadership – “i.e., a pattern of transparent and ethical leader behavior that encourages openness in sharing information needed to make decisions while accepting followers’ inputs” (Avolio et al., 2009, p. 423). Authentic leadership is grounded in positive psychology, it is regarded as a way of leading ethically and truthfully, and it holds the promise of leveraging healthier, happier, and productive workplaces (Avolio et al., 2009). Research about authentic leadership is abundant, with studies showing a positive relationship between authentic leadership behaviors, productivity (Lyubovnikova et al., 2017), in-role performance (e.g., extra role behaviors), job satisfaction, and well-being in the workplace (Walumbwa et al., 2008; Mehmood et al., 2016; Hoch et al., 2018). For nurses, authentic leadership enables trustful relations between leaders and followers (Wong and Cummings, 2009), promotes interpersonal collaboration between peers (Regan et al., 2016), and reduces the frequency of adverse patient outcomes (Wong and Giallonardo, 2013).

However, while previous studies have reported a positive relationship between authentic leadership behaviors and leaders’ ratings of team performance (e.g., Lyubovnikova et al., 2017), empirical evidence that established an empirical link between authentic leadership behaviors and team objective performance indicators, and between authentic leadership behaviors and team subjective performance indicators (as rated by the team leader) are scant. Such scarcity of empirical findings limits our capacity to determine if authentic leadership *can leave up to its promise*, thus preventing theoretical refinement and the development of practical tools that foster authenticity in the workplace (Avolio et al., 2009). What is more, few studies exist that have addressed the relationship between authentic leadership and performance in nurses (e.g., Wong and Laschinger, 2013), or who regarded authentic leadership from a team level perspective (Kellett et al., 2006; Lyubovnikova et al., 2017).

Therefore, the current study aims to integrate the authentic leadership and teamwork literatures by examining the relationship between team leaders’ authentic leadership behaviors and teamwork outcomes in healthcare nurse teams. We do this considering the job demands-resources (JD-R) model, an isomorphic model that can adequately describe individual and team level phenomena in organizations (Bakker and Demerouti, 2017). The current study is then the first to integrate the JD-R model (Bakker and Demerouti, 2017) with the team leadership and the teamwork literatures (e.g., Marques-Quinteiro et al., 2020) to further expand current knowledge on how positive forms of leading can enable effective teamwork in complex work environments such as healthcare.

The JD-R model suggests the bolstering, positive influence of job resources – i.e., the physical, psychological, social, and organizational aspects that enable healthy and productive work (e.g., performing in a psychologically safe team environment) – at the individual and team level of analysis. These resources function as a tool that stimulates personal growth, learning, development and, mainly, they inhibit job demands and the physiological and psychological costs associated with them (Bakker and Demerouti, 2017). Hence, we propose that authentic leadership behaviors could be regarded as a job resource that has a positive relationship with teamwork outcomes, namely, performance and flourishing (Avolio et al., 2009; Laschinger and Fida, 2014), as they regard positive actions that are driven by moral and ethical standards that address team members’ individual and collective needs.

Research by Wong and Laschinger (2013) suggests that nurses who see their supervisors as more authentic (compared to those who do not), report being more satisfied with their job and perceive themselves as having a better performance. Later research by Laschinger and Fida (2014) also suggests that nurses’ supervisor’s authentic leadership behaviors act as resource that mitigates job dissatisfaction and reduces the risk of burnout. Through authentic leadership, team leaders build a positive work environment that is based on authenticity in emotional displays, transparency, and trustworthy relationships and foster self-regulation (Lyubovnikova et al., 2017), making it more likely for team members to engage with the goals and tasks set by their leaders and have a better performance. Furthermore, authentic leadership is associated with flourishing, defined as “the experience of life going well (…) a combination of feeling good and functioning effectively (…) with a high level of mental flourishing, and it epitomizes mental health” (Huppert and So, 2013, p. 838). As authentic leaders expose their team members to positive emotions that lead to optimism, satisfaction, and happiness, it should enable flourishing as an indicator of the well-being of team members (Avolio et al., 2009), Hence, we expect that:

**Hypothesis 1:** Team leaders’ authentic leadership behaviors are positively related with team flourishing (Hypothesis 1a) and team performance (Hypothesis 1b).

Hospital nurses face daily job demands, such as increased workload, role ambiguity, time pressure and sleep deprivation, which ultimately lead to lower levels of nurses’ well-being and patients’ quality care (e.g., Hemingway and Smith, 1999; Laschinger and Fida, 2014; Hofmeyer et al., 2020; Zhang et al., 2020). The JD-R model suggests that job demands are work environment elements forcing individuals to exert continued effort for prolonged periods (e.g., performing physically demanding tasks under high stress; Bakker and Demerouti, 2017). One contextual element of the healthcare environment is the daily bed occupancy, which provides a direct measure of the entire patient load passing through any given healthcare unit or department and is a direct measure of workload (Tarnow-Mordi et al., 2000; Grundmann et al., 2002; Carayon and Gürses, 2005; Chaudhury et al., 2006). In this study we test if daily bed occupancy – as a job demand – influences the extent to which positive leadership behaviors, such as authenticity, can enable effective teamwork. An increase in daily bed occupancy should add more strain to the teamwork of hospital nurses (Carayon and Gürses, 2005). While authentic leadership behaviors are a job resource that builds a positive work environment (which enables successful teamwork and psychological flourishing), the daily bed occupancy can buffer this relationship and act as a contextual stressor, dampening the positivity that results from authentic leadership behaviors. Consequently, the strength of the positive relationship between authentic leadership as a job resource and teamwork outcomes should be less for a higher number of daily bed occupancy as a job demand. We expect that:

**Hypothesis 2:** Team leaders’ authentic leadership behaviors are positively related with team flourishing (Hypothesis 2a) and team performance (Hypothesis 2b). This relationship is stronger when daily bed occupancy is low rather than high.

## Method

### Procedure

Data was collected in March 2017, at two hospitals in one European capital over one month. After obtaining approval from the ethical committees of both hospitals (who also intervene as participants’ representatives), two students visited the hospital facilities and invited nurse teams to complete an individual and anonymous paper and pencil questionnaire (e.g., surveys asked no information that would identify the participants). At each hospital, students met the in-shift head nurse of each healthcare unit to present the study and ask for their team’s participation. All in-shift head nurses consented with the study and gave permission to invite nurses to fill in the survey. Nurses were also asked to read the survey informed consent before they decide if they would enroll. Nurses’ participation was often contingent on the time they had available to complete the paper and pen survey, which means that the students sometimes had to return another time to collect the data.

### Participants

Participants were from 33 nurse teams (*N* = 106 nurses) and their direct supervisors (*N* = 33 head nurses). The age of the participants ranged between 22 and 64 years old (*M* = 35.49, SD = 8.97), with 71.9% being female. Regarding participants educational background, 72.7% (*n* = 101), 15.1% had a degree specialization, and 8.6% had a master’s degree. Team tenure ranged between 1 and 30 years (*M* = 5.58, SD = 6.60). In Hospital 1, team size ranged between 1 and 6 nurses (*M* = 5.15, SD = 2.26). In Hospital 2, team size ranged between 3 and 13 (*M* = 7.21, SD = 2.32).

### Measures

#### Authentic Leadership

Authentic leadership was measured using a 15-item scale developed by Walumbwa et al. (2008). Team members were asked to share the frequency their team leader displayed authentic leadership behaviors, using a Likert-type scale ranging from 1 (Never) to 5 (Very frequently, if not always). An example of one item is “My team leader seeks feedback to improve interactions with others.” Cronbach alpha was0.96.

#### Flourishing

Team flourishing was measured using 8 items from Diener et al. (2010). Team members were asked to share the extent to which they agreed that they experienced flourishing, using a Likert-type scale ranging from 1 (Totally disagree) to 5 (Totally agree). An example of one item is “I am competent and capable in the activities that are important to me.” Cronbach alpha was 0.85.

#### Daily Bed Occupancy

Team leaders were asked to report the number of beds under their responsibility that were occupied (at survey completion), as a measure of daily bed occupancy.

#### Team Performance

Team performance was measured using 3 items developed by Aubé and Rousseau (2005). Team leaders were asked to share to what extent they agreed that their team was effective, using a Likert-type scale ranging from 1 (Totally disagree) to 5 (Totally agree). An example of one item is “The members of this team achieve the goals that were set to them.” Cronbach alpha was 0.88. Since leaders’ ratings provide a subjective measure of team performance, we also asked for an objective measure of team performance. Hence, team leaders were asked to report the number of medical discharges during their shift (at survey completion), as an objective measure of team performance as they signal an improvement in patient health status, which is the primary goal of healthcare teams (Guerlain et al., 2005).

## Results

Before the research hypotheses could be tested at the team level of analysis, the level of agreement between the ratings of team members (rwg) and the intraclass correlation indexes (ICC1 and ICC2) were estimated (James et al., 1993; Bliese, 2000). The rwg for authentic leadership and flourishing were 0.90 and 0.93, respectively; authentic leadership was ICC(1) 0.15 and ICC(2) 0.40; and flourishing was ICC(1) 0.04 and ICC(2) 0.15. Following recommendations by James et al. (1993) and Bliese (2000), the results of the aggregation indexes were regarded as acceptable to justify the aggregation of individual responses to the team level and continue with hypotheses testing. Even though the ICCs for team flourishing were low, Bliese (2000) suggests that this should not be a reason to discard aggregation as the lower ICC values only make hypothesis testing more conservative.

Table 1 reports the descriptive statistics and correlations. The results displayed in the correlation table show that team leaders authentic leadership behaviors were unrelated with team flourishing (*r* = 0.21, *p* = 0.24), team performance (*r* = 0.16, *p* = 0.38), and medical discharges (*r* = −0.003, *p* = 0.99). The lack of a statistically positive and significant correlation between authentic leadership behaviors and the outcome variables rejects Hypotheses 1a and 1b.

**TABLE 1:** Descriptive statistics and correlations.

	*M*	SD	1	2	3	4
1. Team leader authentic leadership.	3.72	0.57	1	–	–	–
2. Bed occupancy.	18.33	16.45	0.29	1	–	–
3. Team flourishing.	4.37	0.28	0.21	0.12	1	–
4. Team performance.	4.30	0.60	16	−0.06	25	1
5. Medical discharges.	0.49	1.00	−0.003	0.09	−0.04	0.14

**N* = 33 teams.*

Hypotheses 2a and 2b were tested using PROCESS for SPSS. The results suggest that daily bed occupancy does not moderate the relationship between team leaders’ authentic leadership and team flourishing, *B* = 0.001, SE = 0.01, *t* = 0.24, *p* = 0.81, 95% CI (−0.009, 0.012). This result rejects Hypothesis 2a.

**FIGURE 1:**
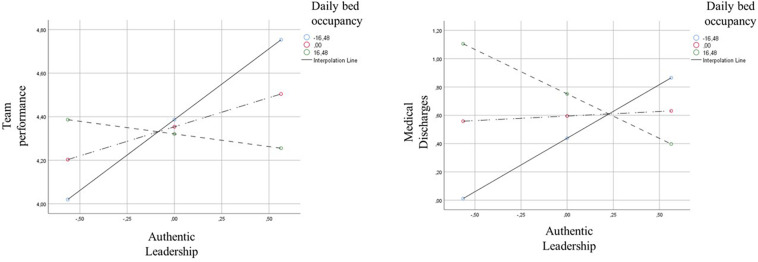
Moderation graphs for the interaction between team leaders’ authentic leadership and daily bed occupancy, regressed on team performance **(left graph)** and medical discharges **(right graph)**.

The results displayed in Table 2 show that daily bed occupancy hinders the relationship between team leaders’ authentic leadership and team performance, *B* = –0.02, SE = 0.01, *t* = −2.23, *p* = 0.033, 95% CI (−0.045, −0.002). This result supports Hypothesis 2b. The interaction graph (left side) displayed in Figure 1 further suggests that the strength of the relationship between the authentic leadership behaviors of nurse team leaders and team performance is stronger at lower levels of daily bed occupancy. When the level of daily bed occupancy increases, authentic leadership behaviors become ineffective to enable good performance.

**TABLE 2:** Moderation results for team performance as the outcome variable.

	*R* ^2^_*change*_		*F*(1,29)		*p*	
			
Model summary for the moderation	0.15		4.99		0.03	
					**95% CI**	
**Main effects and interactions**	**B**	**SE**	***t***	***p***	**Lower limit**	**Upper limit**

Constant	4.35	0.10	83.63	0.00	4.145	4.563
Team authentic leadership	0.27	0.19	1.42	0.17	−0.117	0.651
Bed occupancy	−0.002	0.01	−0.31	0.75	−0.015	0.011
Team authentic leadership^*x*^ Bed occupancy	−0.02	0.01	−2.23	0.03	−0.045	−0.002

**N* = 33 teams. Team authentic leadership and occupied beds were mean centered prior to analysis. Data analysis was performed using 5000 bootstrap samples with a 95% CI. The “x” stands for the interaction.*

Finally, in this study we also collected data on the number of medical discharges as a direct, objective measure of team performance. Like team leaders’ subjective ratings of team performance, the results presented in Table 3 highlight that daily bed occupancy negatively moderates the relationship between team leaders’ authentic leadership and medical discharges, *B* = –0.04, SE = 0.02, *t* = −2.38, *p* = 0.024, 95% CI (−0.078, −0.006). The interaction graph (right side) displayed in Figure 1 further suggests that the strength of the relationship between the authentic leadership behaviors of nurse team leaders and medical discharges is stronger at lower levels of daily bed occupancy. When the level of daily bed occupancy increases, authentic leadership behaviors become ineffective to enable medical discharges.

**TABLE 3:** Moderation results for medical discharges as the outcome variable.

	*R* ^2^_*change*_		*F*(1,29)		*p*	
			
Model summary for the moderation	0.16		5.64		0.02	
					**95% CI**	
**Main effects and interactions**	**B**	**SE**	***t***	***p***	**Lower Limit**	**Upper Limit**

Constant	0.60	0.18	3.43	0.002	0.239	0.949
Team authentic leadership	0.06	0.32	0.20	0.84	−0.587	0.716
Bed occupancy	0.01	0.01	0.87	0.39	−0.013	0.032
Team authentic leadership^*x*^ Bed occupancy	−0.04	0.01	−2.38	0.02	−0.078	−0.006

**N* = 33 teams. Team authentic leadership and occupied beds were mean centered prior to analysis. Data analysis was performed using 5000 bootstrap samples with a 95% CI. The “x” stands for the interaction.*

## Discussion

This study integrated the JD-R model with the team leadership and the teamwork literatures to examine if the relationship between authentic leadership – as a job resource – and teamwork outcomes was sensitive to contextual boundary conditions – the number of occupied beds – as a job demand. The results partially support this hypothesis as they suggest that authentic leadership enables team performance only when the number of occupied beds is low. Additionally, contrary to what was expected, authentic leadership had no positive relationship with team flourishing.

Authentic leadership fosters positive work environments. Hence, studying the relationship between team leadership and teamwork outcomes in unique contexts (such as healthcare) is essential; it can help advance existing knowledge on the boundary conditions that define the relationship between authentic leadership behaviors and team results. Despite recent studies suggesting that authentic leadership enables positive results for individuals and collectives (e.g., Wong et al., 2013; Hoch et al., 2018), for nurses, only when contextual stressors are low does authentic leadership behaviors from the team leader build the expected quality care in the service provided and positive well-being between team members. Hence, as leadership is a process that unfolds in one setting, is sensitive to changes in it, and influences team outcomes (Kozlowski et al., 2009), our findings highlight the importance of context to better understand the relationship between authentic leadership and work-related outcomes.

These results raise some questions on how and in which contexts can an authentic leadership approach be effective and how other leadership approaches could complement authentic leadership to yield more positive organizational outcomes. This study contributes to extend theory on team leadership that has focused more on task-related leadership functions rather than social and psychological state-oriented leadership functions, despite general leadership theories always emphasizing both components (Burke et al., 2006; Avolio et al., 2009). Indeed, there is empirical evidence to demonstrate the importance of social-oriented leadership behaviors (Kozlowski et al., 2009; Morgeson et al., 2010), with leaders listening to team members and being able to manage their needs carefully to integrate those needs and clarify their roles (Graça and Passos, 2015).

Authentic leadership could be conceptualized as one part of social-oriented leadership that has an impact under certain contextual conditions, but other leadership components could be combined with authentic leadership (Hoch et al., 2018) to yield more impactful flourishing and performance results. For example, psychological and well-being states depend highly on feelings of self-efficacy and the team members having the resources and clear goals in place. Research shows that team members have less of an emotional reaction to stressors and, therefore, are in a better psychological state when leaders provide clear team goals, a clear specification of member roles, and unambiguous performance strategies (Zaccaro et al., 2001). This is particularly important in hospital teams, where collaboration and cooperation are enhanced through the definition of the mission and the team identity, the development of shared objectives, and the creation of independent roles for all team members (Landeweerd and Boumans, 1994), leadership actions that can complement authentic leadership behaviors. How leaders set these roles, as well as norms and expectations of interpersonal relationships, can provide direction, motivation, and social integration. This will make it easier to monitor the responses of team members, facilitating future team flourishing and performance. Moreover, specifically in high pressure situations where urgent decisions need to be made, setting goals, providing timely feedback and other task related functions can complement authentic leaders focusing solely on listening carefully to the ideas of others, and not emphasizing their own point of view at the expense of others, behaviors that can be counterproductive to make urgent decisions under high pressure (Sanchez-Manzanares et al., 2020).

Future studies can seek to combine authentic leadership approaches with task-related functions, to see an increase of both affective and objective team outcomes. Future studies could also build on the limitations of this research to test the robustness of our findings and expand them. First, the cross-sectional design in our study can raise concerns about common-method variance and assumptions of causality (Spector, 2019). This could be solved by adopting a temporal data collection approach, where each variable is collected on a different occasion (e.g., one per day; one per week). Alternatively, a longitudinal design could shed light on the temporal dynamics of team leadership and team outcomes, and how these are shaped by context. Second, future studies could also adopt a multilevel research design to test the extent to which contextual features of the nurses’ task environment (including bed occupancy rates), shape how team leadership behaviors collective and individual outcomes (e.g., performance and well-being). Third, the sample size of *n* = 33 is borderline for the determination of robust moderation effects, which leads us to ask researchers and practitioners to be careful in the generalization of these findings (Marôco, 2018). Future studies should try to replicate our findings using larger sample sizes that allow for more robust assumptions about the relationship between team authentic leadership, bed occupancy rates, team flourishing, and team performance. Finally, the performance of hospital teams is not easily assessed, especially because performance outcome indicators such as infection rate after surgery, patient mortality, and hospital readmissions cannot always be directly related with the quality of team processes (Neuman et al., 2014; Lyubovnikova et al., 2015; Schmutz et al., 2019). As an example, a team can deliver a specific treatment protocol with 100% accuracy, and the patient health condition still worsens. In the case of medical discharges, this might also be a lengthy process, that is dependent on clinical, bureaucratic, and social factors that can override teamwork. Therefore, future studies could consider the adoption of infection rates after treatment or surgery, as well as patient readmission as alternative measures of team performance.

## Conclusion

To conclude, the outcomes of our research can be regarded by practitioners aiming to enhance their teams’ performance (especially in healthcare), should be mindful that positive, authentic leadership behaviors enable better team performance in healthcare, except when context adversity (e.g., bed occupancy rate) undermines the authentic leadership behaviors. What is more, leading authentically might not be enough to build positive states in nurse teams.

## Data Availability Statement

The raw data supporting the conclusions of this article will be made available by the authors, without undue reservation.

## Ethics Statement

The studies involving human participants were reviewed and approved by the Comissão de Ética para a Saúde, Hospital Lusíadas Lisboa. The patients/participants provided their written informed consent to participate in this study.

## Author Contributions

PM-Q and AG wrote the manuscript. PM-Q and DM performed the analysis. FC reviewed the manuscript. DM collected the data. All authors contributed to the article and approved the submitted version.

## Conflict of Interest

The authors declare that the research was conducted in the absence of any commercial or financial relationships that could be construed as a potential conflict of interest.

## Publisher’s Note

All claims expressed in this article are solely those of the authors and do not necessarily represent those of their affiliated organizations, or those of the publisher, the editors and the reviewers. Any product that may be evaluated in this article, or claim that may be made by its manufacturer, is not guaranteed or endorsed by the publisher.
